# Mean Platelet Volume-to-Platelet Ratio as a Preoperative Marker for Distinguishing Pancreatic Cancer and Chronic Pancreatitis

**DOI:** 10.7759/cureus.111354

**Published:** 2026-06-23

**Authors:** Murshidul Arefin, Md. Kawsar Bhuiyan, Anupam Debnath, Golam Mahmud Rayhan, Sabuj Kumar Patra, Nur Alam Mohim, Mohammad Saief Uddin, Salma A Tohfa, Amit Chowdhury, Shah Md. Ezaz-ul Haque

**Affiliations:** 1 Department of Hepatobiliary, Pancreatic and Liver Transplant Surgery, Bangladesh Medical University, Dhaka, BGD; 2 Department of Surgery, National Gastroliver Institute and Hospital, Dhaka, BGD; 3 Department of Surgery, Evercare Hospital Dhaka, Dhaka, BGD; 4 Department of Surgery, Directorate General of Health Services, Dhaka, BGD; 5 Department of Surgery, Rupganj Upazila Health Complex, Dhaka, BGD; 6 Department of Surgery, Bangladesh Medical University, Dhaka, BGD; 7 Department of Surgery, Popular Medical College and Hospital, Dhaka, BGD; 8 Department of Community Medicine, Dhaka Medical College, Dhaka, BGD

**Keywords:** ca 19-9, chronic pancreatitis, hepatobilliary, mean platelet volume-to-platelet ratio, pancreatic cancer

## Abstract

Background

Differentiating pancreatic cancer from chronic pancreatitis remains clinically challenging due to the overlap of symptoms, biochemical abnormalities and imaging characteristics. Carbohydrate antigen 19-9 (CA 19-9) is a very common marker employed in the assessment of pancreatic cancer but is not sufficiently specific because it has been shown to be elevated in cases of inflammatory and obstructive pancreaticobiliary disease. A new and easy-to-obtain platelet-derived inflammatory marker, mean platelet volume to platelet ratio (MPR), may provide further utility in the differentiation of malignant and benign inflammatory pancreatic disease.

Objective

We aimed to assess the MPR value in the diagnosis of pancreatic cancer versus chronic pancreatitis in patients undergoing pancreatic surgery.

Materials and methods

This was a prospective diagnostic accuracy study carried out in the Department of Hepatobiliary, Pancreatic and Liver Transplant Surgery, Bangladesh Medical University, Dhaka, from September 2023 to August 2024. Adult patients who underwent pancreatic surgery for suspected pancreatic cancer or chronic pancreatitis were included. The final histopathological diagnosis of the surgical specimen was used as the reference standard. Patients who had an acute medical condition or psychiatric condition were excluded. A total of 35 patients were included (18 with pancreatic cancer and 17 with chronic pancreatitis). A structured checklist was used to obtain demographic, clinical and laboratory data. Preoperative laboratory parameters within seven days of surgery were used to calculate MPR, which is defined as mean platelet volume (MPV) divided by platelet count. The accuracy of diagnosis was determined by receiver operating characteristic curve analysis.

Results

There was no significant difference between the two groups in terms of age, sex, or body mass index (BMI). Chronic pancreatitis was significantly more likely to have endocrine and exocrine insufficiency. Significantly elevated concentrations of serum amylase, lipase, MPV, MPR, total bilirubin and CA 19-9, along with decreased platelet counts, were seen in individuals with pancreatic cancer. Chronic pancreatitis was more often associated with diabetes mellitus, whereas an American Society of Anaesthesiologists (ASA) score of 1 was associated with pancreatic cancer. MPR demonstrated high diagnostic performance, with an AUC of 0.908, sensitivity of 83.3%, specificity of 100.0%, a Youden index of 0.833, and a cutoff value of 0.053. MPV showed good discriminatory performance, CA 19-9 showed fair discriminatory performance, platelet count showed strong inverse discriminatory ability, and CEA had little diagnostic utility.

Conclusions

MPR may serve as a low-cost and easily available adjunctive preoperative marker for differentiating pancreatic cancer from chronic pancreatitis; however, its cutoff value and clinical applicability require validation in larger, multicentre prospective cohorts.

## Introduction

Pancreatic ductal adenocarcinoma (PDAC) mainly develops from the exocrine pancreas and is the most common pancreatic cancer, which represents over 90% of exocrine pancreatic cancer [1,2]. Despite ranking 10th in global cancer incidence, PDAC is the seventh leading cause of cancer-related mortality owing to its poor prognosis [3]. In Bangladesh, the age-related standardised mortality rate (ASMR) from pancreatic cancer was estimated to be around 1.15 per 100,000 population based on the data from the World Health Organization (WHO) [4]. Diagnosing the disease early, when it is localised, offers better chances for successful surgery, but most patients are diagnosed at an advanced stage and are treated primarily for palliation [5,6]. Chronic pancreatitis (CP) is a non-cancerous inflammatory condition of the pancreas that is painful, leads to fibrosis, and causes irreversible damage to the tissue [7]. Inflammatory pancreatic masses and pancreatic cancer can have identical clinical symptoms, such as abdominal pain, weight loss, jaundice, and imaging findings, and it can be difficult to differentiate between them in everyday practice [8,9]. Clinically, this diagnostic uncertainty is significant, since it can delay potentially curative surgery if cancer is not recognised promptly, and it may lead to unnecessary invasive procedures if patients have a benign disease [10]. The current diagnostic technologies are useful, but not perfect. Carbohydrate antigen 19-9 (CA 19-9) is the most commonly used serum marker but is limited in the early stages of the disease and may also be elevated in other benign conditions like pancreatitis and obstructive jaundice [11]. These tests are routinely performed, including ultrasonography, contrast-enhanced computed tomography (CECT) and magnetic resonance imaging (MRI); however, inflammatory masses and PDAC can be difficult to distinguish radiologically [12]. Other markers, such as immunoglobulin G4 (IgG4) and CA 19-9, have been studied, but none have proven to be useful as a single diagnostic test [13]. Blood-based indices are therefore the focus of interest as simple and readily available diagnostic and prognostic parameters in cancer [14]. The platelet size and activity measured by mean platelet volume (MPV) have been used in diagnosing malignancies [15]. The mean platelet volume to platelet ratio (MPR) is a combination of platelet activation and platelet count and has been clinically important in colorectal cancer and pancreatic disease [16,17]. Elevated MPR has been previously associated with low survival rates [17], and an increase in MPR in pancreatic cancer compared to benign pancreatic disease is reported [17]. Similar prognostic relationships have been reported in hepatocellular, lung, oesophageal and colorectal cancers [18-21]. The biological rationale for using MPR is that it combines two platelet-related changes: increased MPV, reflecting platelet activation, and reduced platelet count, which may reflect platelet consumption in malignancy-associated inflammation and a prothrombotic state. Therefore, the ratio may capture platelet activation and platelet consumption more comprehensively than either MPV or platelet count alone. Previous studies have evaluated MPR in pancreatic cancer and mass-forming CP [17]; however, evidence remains limited, particularly in surgically confirmed cohorts and in comparison with conventional markers such as CA 19-9, CEA, MPV, and platelet count. The present study, therefore, aimed to assess the diagnostic value of preoperative MPR for differentiating pancreatic cancer from CP in a histopathologically confirmed surgical cohort.

## Materials and methods

Study design and setting

This was a prospective diagnostic accuracy study carried out at the Department of Hepatobiliary, Pancreatic and Liver Transplant Surgery, Bangladesh Medical University (BMU), Shahbag, Dhaka, Bangladesh, following Institutional Review Board (IRB) approval between September 2023 and August 2024.

Study participants

Deploying a purposive sampling technique, adults who had undergone pancreatic surgery for suspected pancreatic cancer and CP were selected. No formal a priori sample-size calculation was performed. This was an exploratory study based on the available number of eligible surgically treated patients during the study period. Therefore, the stability of ROC-derived estimates, including cutoff values, sensitivity, and specificity, should be interpreted cautiously and validated in larger prospective cohorts. A total of 35 patients (18 patients with pancreatic cancer and 17 patients with CP) were included in the sample. Enrolment criteria for study participants are mentioned in Table 1.

**Table 1:** Enrolment criteria for study participants

Inclusion criteria	Exclusion criteria
Adult patients (age ≥ 18 years) of either sex	Patients for whom surgical treatment was not required
Patients with either chronic pancreatitis or pancreatic cancer (PC) and undergoing surgery with suggestive imaging before surgery	Patients with acute medical illness
Patients who were histopathologically proven to have PC or chronic pancreatitis (CP)	Patients suffering from psychiatric disorders

Study variables

Demographic factors were age, sex and body mass index (BMI). Clinical variables recorded were diabetes mellitus, hypertension and American Society of Anaesthesiologists (ASA) score. Laboratory parameters consisted of complete blood count, MPV, platelet count, MPR, carcinoembryonic antigen (CEA), CA 19-9, serum albumin, serum bilirubin, C-reactive protein, serum amylase, and serum lipase. MPR was defined as the ratio of the MPV/MPV to the platelet count. Laboratory values were taken from diagnostic tests performed within seven days before surgery.

Outcome or reference standard

The final histopathological diagnosis of the surgical specimen was used as the reference standard, and the patients were categorised into CP and pancreatic cancer groups. The diagnostic accuracy of MPR was compared with histopathological diagnosis and other markers, such as CA 19-9, CEA, platelet count and MPV. Formal blinding of the pathologists to preoperative platelet indices and MPR could not be confirmed, and this has been acknowledged as a methodological limitation.

Data collection procedure

A structured checklist was used to collect data from medical records, history taking, physical examination, biochemical markers, preoperative assessment, intraoperative findings, histopathology reports and postoperative assessment. Physical status was evaluated using the ASA score on a scale of 1-6 [22]. All participants gave verbal and written informed consent.

Definitions

MPR was defined as a platelet-derived inflammatory marker which could be used to differentiate malignant from benign pancreatic disease [17]. CP was considered a fibro-inflammatory pancreatic syndrome characterised by a persistent inflammatory response to parenchymal damage or stressors. The definition of pancreatic cancer was a malignant epithelial tumour of the pancreas, most commonly PDAC [23].

Ethical considerations

The Institutional Review Board of BMU provided ethical clearance. Participants were made aware of the study in the local language. Confidentiality and anonymity were preserved, and non-participation did not impact the quality of care received.

Statistical analysis

The data were entered in Microsoft Excel 2019 (Microsoft® Corp., Redmond, WA) and analysed in SPSS (IBM SPSS Statistics for Windows, IBM Corp., Version 26, Armonk, NY). Categorical data were expressed using frequencies and percentages and compared using the chi-square or Fisher's exact test. Normally distributed continuous data were expressed as mean ± SD or median (IQR) when not normally distributed and compared with Student's t-test or the Mann-Whitney U test where appropriate. MPR, CA 19-9, CEA, platelet count, and MPV were analysed by ROC curves. A P < 0.05 value was considered statistically significant. Because this was an exploratory study, P-values were not adjusted for multiple comparisons. Therefore, findings with borderline statistical significance should be interpreted cautiously and validated in larger studies.

## Results

A total of 35 patients were included, consisting of 18 with pancreatic cancer and 17 with CP. The mean ± SD age in the pancreatic cancer group was greater than in the CP group but not statistically significant (46.4 ± 13.82 vs. 39.82 ± 9.27 years, P = 0.108). There was no significant difference between the groups in terms of sex and BMI distribution (P = 0.508 and P = 0.053, respectively). The proportion of endocrine insufficiency was also much higher among the CP group compared to the pancreatic cancer group (100% vs. 61%, P = 0.008). Similarly, exocrine insufficiency was prevalent in CP (88% vs. 11%, P < 0.001). There was no significant difference between the groups in terms of the type of surgical procedure (P = 0.148) (Table 2).

**Table 2:** Comparison of baseline data between the pancreatic cancer group and the chronic pancreatitis group (N = 35) Values are expressed as n (%) unless otherwise indicated. BMI: body mass index; CA 19-9: carbohydrate antigen 19-9; CEA: carcinoembryonic antigen; CRP: C-reactive protein; fL: femtoliter; IQR: interquartile range; LPJ: lateral pancreaticojejunostomy; MPR: mean platelet volume-to-platelet ratio; MPV: mean platelet volume; SD: standard deviation; U/L: units per litre

Variable	Total (N = 35)	Pancreatic cancer (n = 18)	Chronic pancreatitis (n = 17)	Test used/statistic/effect size	P-value
	N (%)	n (%)	n (%)		
Age (years), mean (SD)	43.23 (12.14)	46.4 (13.82)	39.82 (9.27)	Student's t-test; t(33) = 1.654; Cohen's d = 0.559	0.108
Sex					
Female	18 (51)	8 (44)	10 (59)	Chi-square test; (χ²(1, N = 35) = 0.724; Cramer's V = 0.144)	0.508
Male	17 (49)	10 (56)	7 (41)	-	-
BMI (kg/m²)					
<18.5	14 (40)	4 (22)	10 (59)	Exact test	0.053
18.5 to 22.9	19 (54)	12 (67)	7 (41)	-	-
≥23	2 (6)	2 (11)	0 (0)	-	-
Endocrine insufficiency					
Yes	28 (80)	11 (61)	17 (100)	Fisher's exact test	0.008
No	7 (20)	7 (39)	0 (0)	-	-
Exocrine insufficiency					
Yes	17 (49)	2 (11)	15 (88)	Chi-square test; (χ²(1, N = 35) = 20.818; Cramer's V = 0.771)	<0.001
No	18 (51)	16 (89)	2 (12)	-	-
Type of procedure					
Roux-en-Y LPJ	5 (43)	0 (0)	5 (88)	Exact test	0.148
Frey’s procedure	2 (6)	0 (0)	2 (12)	-	-
Whipple’s Procedure	13 (37)	13 (72)	0 (0)	-	-
Operative palliation of pancreatic cancer	5 (14.0)	5 (28.0)	0 (0.0)	-	-
Baseline laboratories					
Serum amylase (U/L), median (IQR)	18.0 (30.0)	18.0 (38.0)	14.0 (19.0)	Mann-Whitney U test; U = 81.0; rank-biserial r = 0.471	0.015
Serum lipase (U/L), median (IQR)	17.0 (24.0)	25.50 (22.0)	6.0 (9.0)	Mann-Whitney U test; U = 32.0; rank-biserial r = 0.791	<0.001
CRP (mg/L), median (IQR)	0.02 (4.71)	0.025 (0.75)	0.020 (5.01)	Mann-Whitney U test; U = 121.0; rank-biserial r = 0.209	0.276
Haemoglobin, g/dL, mean (SD)	11.19 (1.35)	11.0 (1.44)	11.43 (1.26)	Student's t-test; t(33) = -0.998; Cohen's d = -0.338	0.325
Platelet count (×10³ cells/µL), median (IQR)	230 (150.0)	190 (43.0)	310 (115.0)	Mann-Whitney U test; U = 30.0; rank-biserial r = -0.804	<0.001
MPV (fL), mean (SD)	10.76 (1.48)	11.6 (1.52)	9.89 (0.80)	Student's t-test; t(33) = 4.10; Cohen's d = 1.386	<0.001
MPR (fL/10³ cells/µL), median (IQR)	0.04 (0.03)	0.06 (0.01)	0.03 (0.01)	Mann-Whitney U test; U = 28.0; rank-biserial r = 0.817	<0.001
Serum total bilirubin (mg/dL), median (IQR)	0.7 (4.04)	3.4 (10.38)	0.7 (0.22)	Mann-Whitney U test; U = 73.0; rank-biserial r = 0.523	0.008
Serum albumin (g/L), median (IQR)	38.0 (11.0)	34.3 (14.5)	42.0 (10.5)	Mann-Whitney U test; U = 111.5; rank-biserial r = -0.271	0.168
CEA (ng/mL), median (IQR)	3.01 (2.96)	2.45 (3.92)	3.16 (2.37)	Mann-Whitney U test; U = 152.0; rank-biserial r = -0.003	0.987
CA 19-9 (U/mL), median (IQR)	98.0 (231.08)	183.09 (647.20)	17.80 (99.15)	Mann-Whitney U test; U = 69.0; rank-biserial r = 0.549	0.006

Baseline laboratory findings

The baseline laboratory findings indicated that serum amylase was significantly higher in the pancreatic cancer group than in the CP group, with median values of 18.0 U/L versus 14.0 U/L, respectively, P = 0.015; median lipase: 25.50 vs. 6.0 U/L, P < 0.001. Conversely, the levels of C-reactive protein were similar between the two groups, with no statistically significant difference, P = 0.276. Haemoglobin, serum albumin, and CEA levels were not significantly different between the groups, with P = 0.325, P = 0.168, and P = 0.987, respectively. Compared with the CP group, the pancreatic cancer group had significantly lower platelet counts (median: 190 × 10³ cells/µL vs. 310 × 10³ cells/µL, P < 0.001) and significantly higher MPV (11.6 ± 1.52 vs. 9.89 ± 0.80 fL, P < 0.001), MPR (median: 0.06 vs. 0.03, P < 0.001), serum total bilirubin (median: 3.4 mg/dL vs. 0.7 mg/dL, P = 0.008), and CA 19-9 levels (median: 183.09 U/mL vs. 17.80 U/mL, P = 0.006) (Table 2).

Clinical characteristics

Anaemia was not significantly associated with pancreatic cancer (P = 0.999). Diabetes mellitus was significantly less common in the pancreatic cancer group than in the CP group (61% vs. 94%; OR: 0.10, 95% CI: 0.01-0.92; P = 0.041). Although hypertension was more frequent in the pancreatic cancer group, the association was not statistically significant (33% vs. 6%; OR: 8.0, 95% CI: 0.85-75.56; P = 0.088). An ASA score of 1 was significantly more common in the pancreatic cancer group (39% vs. 6%; OR: 10.18, 95% CI: 1.09-94.83; P = 0.041). However, this finding should be interpreted cautiously because it may reflect selection or classification bias in this surgically managed cohort rather than a true disease-related biological association (Table 3).

**Table 3:** Analysis of clinical characteristics between the pancreatic cancer group and the chronic pancreatitis group (N = 35) ASA: American Society of Anesthesiologists; CI: confidence interval; OR: odds ratio; n (%): number (percentage) Test used: Fisher's exact test for anaemia, diabetes mellitus, hypertension, and ASA score. For Fisher's exact tests, ORs with 95% CIs served as effect estimates.

Characteristics	Total (N = 35), N (%)	Pancreatic cancer (n = 18), n (%)	Chronic pancreatitis (n = 17), n (%)	OR (95% CI)	P-value
Anaemia					
Present	5 (14)	3 (17)	2 (12)	1.50 (0.22-10.31)	0.999
Absent	30 (86)	15 (83)	15 (88)	-	-
Diabetes mellitus					
Present	27 (77)	11 (61)	16 (94)	0.10 (0.01-0.92)	0.041
Absent	8 (23)	7 (39)	1 (6)	-	-
Hypertension					
Present	7 (20)	6 (33)	1 (6)	8.0 (0.85-75.56)	0.088
Absent	28 (80)	12 (67)	16 (94)	-	-
ASA score					
1	8 (23)	7 (39)	1 (6)	10.18 (1.09-94.83)	0.041
≥2	27 (77)	11 (61)	16 (94)	-	-

Analysis of the ROC curve and cutoff points

Among the assessed biomarkers, MPR showed the best discriminatory performance for differentiating pancreatic cancer from CP, with a cutoff value of 0.053, sensitivity of 83.3%, specificity of 100.0%, Youden index of 0.833, and AUC of 0.908 (95% CI: 0.790-0.999; P < 0.001). MPV also showed good diagnostic performance, with a cutoff of 10.6, sensitivity of 77.8%, specificity of 88.2%, and AUC of 0.842 (95% CI: 0.702-0.981). CA 19-9 showed fair diagnostic accuracy, with a cutoff of 79.50, sensitivity of 77.8%, specificity of 70.6%, and AUC of 0.775 (95% CI: 0.615-0.934; P = 0.006). Platelet count showed inverse discriminatory performance because lower platelet counts were associated with pancreatic cancer in this cohort. Therefore, the AUC should be interpreted after correcting for directionality. The inverted AUC is approximately 0.902, suggesting strong inverse discriminatory ability. The previously reported cutoff of 421.0 × 10³ cells/µL should not be interpreted as a clinically meaningful threshold without reanalysis using the correct predictor direction (Table 4).

**Table 4:** Cutoff values of pre-surgical markers used to differentiate pancreatic cancer from chronic pancreatitis: sensitivity, specificity, Youden index, and AUC AUC: area under the receiver operating characteristic curve; CA 19-9: carbohydrate antigen 19-9; CEA: carcinoembryonic antigen; CI: confidence interval; MPR: mean platelet volume-to-platelet ratio; MPV: mean platelet volume

Variable	Cutoff	Sensitivity	Specificity	Youden index	AUC	95% CI	P-value
Platelet count (×10³ cells/µL)	421.0	11.1	82.4	- 0.065	0.098	0.0-0.228	<0.001
MPV (fL)	10.6	77.8	88.2	0.660	0.842	0.702-0.981	0.001
MPR (fL/10³ cells/µL)	0.053	83.3	100.0	0.833	0.908	0.790-0.999	<0.001
CA 19-9 (U/mL)	79.50	77.8	70.6	0.484	0.775	0.615-0.934	0.006
CEA (ng/mL)	1.92	66.7	23.5	-0.098	0.498	0.297-0.700	0.987

**Figure 1:**
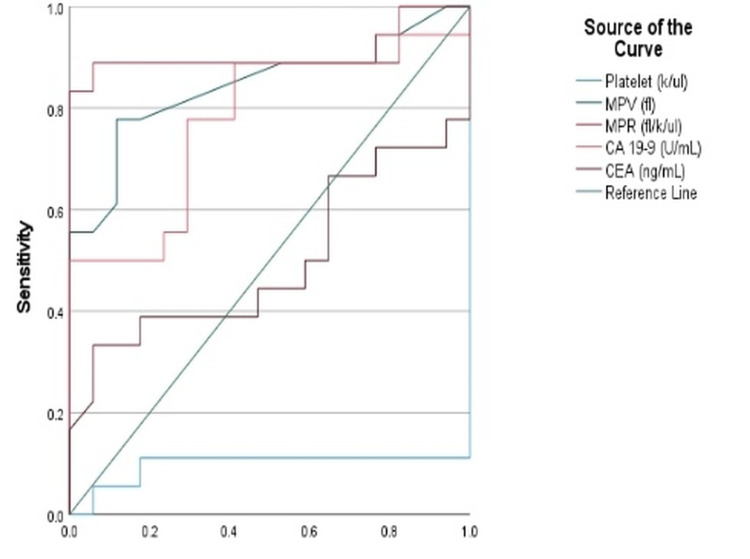
Receiver operating characteristic curve of different biomarkers for differentiating pancreatic cancer from chronic pancreatitis (N = 35) CA 19-9: carbohydrate antigen 19-9; CEA: carcinoembryonic antigen; MPR: mean platelet volume-to-platelet ratio; MPV: mean platelet volume; ROC: receiver operating characteristic

Figure 1 shows the ROC curves of the evaluated biomarkers. MPR had the highest diagnostic accuracy, followed by MPV and CA 19-9, whereas platelet count and CEA demonstrated limited diagnostic utility.

## Discussion

The present study assessed clinically relevant differences in pancreatic cancer and CP and found several significant results that could be used to improve the discrimination between these two challenging entities before surgery. Amylase, lipase, MPV, MPR, total bilirubin, and CA 19-9 levels were significantly higher, while platelet count was lower, in the pancreatic cancer group, while endocrine/exocrine insufficiency and the number of cases of diabetes mellitus were higher in the CP group. After accounting for ROC directionality, platelet count also demonstrated strong inverse discriminatory performance. Therefore, the present study does not claim that MPR is statistically superior to platelet count. Rather, MPR and inverse platelet count both showed high discriminatory ability in this exploratory cohort. Because no formal pairwise ROC comparison was performed, claims of superiority should be avoided.

The elevated MPR in pancreatic cancer further confirms the importance of platelet-mediated inflammatory markers in malignant pancreatic diseases. Increased MPV levels were reported by Gong et al. in pancreatic cancer, together with an elevated MPV/platelet count ratio, and a higher MPV/platelet count ratio was correlated with shorter overall survival, suggesting the relationship between platelet activation and consumption and the aggressiveness of the tumour [24]. Similarly, Wang et al. suggested that the MPR was significantly different between pancreatic cancer and mass-forming CP and suggested that MPR could be a new independent parameter for distinguishing the two diseases [17]. Therefore, the results are confirmatory of the usefulness of MPR in differentiating malignant from inflammatory pancreatic masses.

This is biologically plausible, as there was a higher MPV and lower platelet count in pancreatic cancer. The systemic inflammatory state, as well as cytokine signalling and immune dysregulation, is associated with pancreatic cancer, and these processes could have the potential to affect platelet turnover and activation [25,26]. Tanţău et al. demonstrated that there was a higher inflammatory profile of pancreatic adenocarcinoma than CP, with differences in serum CRP, IL-6, IL-10 and IL-17 [27]. The reason for this is explained by these results, as an increase in MPV and MPR can be seen as a sign of the malignancy's inflammatory and procoagulant environment.

The performance of MPR for ROC analysis was excellent with an AUC of 0.908, sensitivity of 83.3%, specificity of 100.0% and a cutoff value of 0.053. In Wang et al., the AUC gap between MPR and CA 19-9 was larger than in the current study, which may reflect differences in sample size and disease spectrum [17]; however, this difference could be attributed to the different sample sizes and disease spectrum. Other markers, such as CA 19-9, are also elevated in pancreatic cancer and are useful but may also be elevated in inflammatory/obstructive disease and should not be used alone but in conjunction with MPR and MPV [17,27].

A higher level of bilirubin is seen in pancreatic cancer and may represent malignant biliary obstruction, as seen on cross-sectional imaging and/or endoscopy [12,28]. Ruan et al. reported that blood-based markers complement the computed tomography (CT), MRI, and positron emission tomography/computed tomography (PET-CT) features to better differentiate pancreatic cancer from mass-forming CP [29], and these results indicated that blood-based markers could further enhance this pathway. Endocrine/exocrine insufficiency as a consequence of CP and pain is classified as long-term damage to the gland [8]. Elevated pancreatic enzymes can occur in both pancreatic cancer and CP and should be interpreted in the context of the full clinical picture, imaging findings, and other laboratory markers rather than in isolation [12,17,25].

There are a number of strengths of this study. To begin with, it narrowed down to only the major findings and emphasised markers that are cheap, accessible, and can be measured prior to treatment. Second, it directly compared pancreatic cancer with CP, the clinically relevant differential diagnosis, as opposed to a comparison with healthy controls. Third, it took into account laboratory biomarkers with symptom-based and clinical variables, and the interpretation was more pragmatic as compared to a one-marker method. Lastly, the observation of the highest level of performance of MPR as the biomarker in this study population is consistent with the increasing interest in platelet-based inflammatory indices and in line with recent findings regarding the potential diagnostic and prognostic role of systemic inflammation in pancreatic cancer [17].

There are limitations to this study as well. Small sample size constrains statistical power and possibly also leads to unstable estimates, especially of ROC-derived cutoffs and odds ratios. The single-centre location can provide less generalisability, and there can be selection bias as the study included only surgically managed patients. Patients with advanced unresectable pancreatic cancer, medically managed CP, or indeterminate pancreatic masses in outpatient settings were not represented. Certain major clinical relationships, including ASA score 1 being more prevalent in the pancreatic cancer group, might be due to the sampling nature, as opposed to the disease biology. Moreover, the research failed to incorporate imaging results, endoscopic ultrasound (EUS)-guided tissue diagnosis, and cytokine measurements that have a significant role in the contemporary pancreatic mass assessment [12,28,29]. These made the current findings be considered hypothesis-generating. The optimal cutoff values were derived from the same dataset used to assess diagnostic performance. Because no internal bootstrap validation or external validation cohort was used, these cutoff values may be overfit and should be considered preliminary. The discriminatory performance of MPR should be confirmed in larger prospective studies to find out the best cutoffs and whether integrating MPR with CA 19-9 and imaging would enhance real-life diagnostic accuracy.

## Conclusions

This exploratory study suggests that MPR holds potential as an adjunctive preoperative marker for differentiating pancreatic cancer from CP. Raised MPV, MPR, bilirubin and CA 19-9 were associated with pancreatic cancer, as was low platelet count, whereas endocrine and exocrine insufficiency were more characteristic of CP. However, because of the small sample size, single-centre surgical cohort, internally derived cutoff value, and absence of external validation, the findings should be considered preliminary.
